# *Glutamicibacter soli* JF_198 stimulates the rhizosphere colonization of indigenous *Paenibacillus* sp. to suppress cucumber Fusarium

**DOI:** 10.3389/fmicb.2026.1843711

**Published:** 2026-05-26

**Authors:** Yang Jiao, Yiming Liu, Qixiong Gao, Liqun Song, Fuxin Sun, Yue Li, Heng Li, Songwei Li, Jingjing Li, Jianfeng Du, Chengfei Li, Xueliang Tian, Runqiang Liu

**Affiliations:** 1 School of Plant Protection and Environment, Henan Institute of Science and Technology, Xinxiang, Henan, China; 2 School of Animal Science and Technology, Northwest A&F University, Yangling, Shaanxi, China; 3 Microbial Research Institute of Liaoning Province, Chaoyang, China; 4 College of Agronomy, Shandong Agricultural University, Tai'an, China

**Keywords:** cucumber fusarium wilt, disease suppression, *Glutamicibacter soli*, interspecific, *Paenibacillus* sp., resident microbiota

## Abstract

**Introduction:**

Beneficial microorganisms can facilitate the formation of advantageous microbial communities in the plant rhizosphere, thereby promoting plant health. Our research indicates that *Glutamicibacter soli* JF_198 can mitigate the severity of cucumber Fusarium wilt caused by *Fusarium oxysporum* f. sp. *cucumerinum*. This study investigates the tripartite association among *Glutamicibacter soli* JF_198, the cucumber rhizosphere microbiota, and cucumber Fusarium wilt suppression.

**Methods:**

We evaluated JF_198-mediated disease suppression in cucumber pot experiments and analyzed rhizosphere bacterial communities by 16S rRNA amplicon sequencing. Rhizosphere transplantation, bacterial isolation, antagonistic screening, and growth, competition, and root colonization assays were performed to assess the role of candidate Paenibacillus isolates under cucumber root-exudate conditions.

**Results:**

JF_198 treatment was associated with changes in selected rhizosphere bacterial taxa, particularly an increased trend in *Paenibacillus*-related taxa. Rhizosphere transplantation experiments showed that JF_198-associated rhizosphere microbiota could partially transfer disease-suppressive effects to sterile soil. We identified *Paenibacillus*-assigned OTU1077 as a candidate indigenous beneficial taxon showing increased relative abundance under JF_198 treatment. Further investigation suggested that JF_198 treatment was associated with enhanced *Paenibacillus* sp. colonization under root-exudate conditions.

**Discussion:**

Our findings suggest that *G. soli* JF_198-mediated disease suppression under controlled pot conditions is associated with changes in selected rhizosphere bacterial taxa and increased relative abundance of indigenous biocontrol-associated bacteria. This study provides preliminary insights into microbiome-associated disease suppression under controlled pot conditions and may inform future development of microbial consortia, pending further validation under field conditions.

## 1 Introduction

In recent years, cucumber Fusarium wilt, caused by *F. oxysporum* f. sp. *cucumerinum,* has emerged as a significant challenge in cucumber cultivation areas, which has been exacerbated by global climate change and monoculture practices (Bartholomew et al., 2022). Currently, the primary control methods for this disease rely heavily on chemical pesticides. However, the limited variety of fungicides is not the cause of pathogen resistance. Furthermore, four physiological races of *F. oxysporum* f. sp. *cucumerinum* have been identified: race 1 (USA), race 2 (Israel), race 3 (Japan), and race 4 (China) (Armstrong et al., 1978; Wang, 2008). However, long-term use of chemical pesticides may have adverse effects on the environment, leading to pesticide residues and fungicide resistance (Lecomte et al., 2016). Biological control, through the use of antagonistic microorganisms (including bacteria, fungi, and actinomycetes), provides a better alternative to chemical control (Alabouvette et al., 2009). Therefore, there is an urgent need to discover new microbial resources to develop effective control measures against this disease.

*Glutamicibacter* sp. plays a crucial ecological role, promoting plant growth and enhancing stress tolerance (Qin et al., 2018). *Glutamicibacter halophytocota* KLBMP 5180-produced exopolysaccharides (EPSs) effectively ameliorated NaCl stress in tomato plants (Chen et al., 2023). *Glutamicibacter* sp. serves as an effective biological control agent, enhancing disease suppression efficacy while inducing plant resistance to pathogens. For instance, *Glutamicibacter* sp. FBE-19 has been demonstrated to be an effective biological control agent against *Erwinia tracheiphila*, which causes cucurbit bacterial wilt, and the genomic data of *Glutamicibacter* sp. FBE-19 reveal multiple biosynthetic gene clusters related to plant growth and disease resistance (Fu et al., 2021).

Biocontrol consortia consisting of multiple microbial strains can enhance the stability and efficacy of disease suppression. These biocontrol consortia can enhance their function by promoting mutual growth and metabolic activities, enabling highly cooperative microbial communities to thrive and maintain their ecological function (Luo et al., 2025). For example, the synergistic effect of *Pseudomonas aeruginosa* and *Burkholderia gladioli* mitigated Cd-induced physiological damage in tomato (Khanna et al., 2019). Microbial interactions play a key role in isolating and constructing beneficial microbial communities from complex environments and introducing them into soil ecosystems (Trabelsi and Mhamdi, 2013). *Bacillus velezensis* SQR9 stimulates resident rhizosphere *Pseudomonas stutzeri* for cucumber health through metabolic interactions (Sun et al., 2022). However, biocontrol bacteria often face challenges in achieving desired outcomes in practical applications. This is largely attributed to complex interspecies interactions that may compromise strain viability and functionality (Wagemans et al., 2022). Therefore, analyzing the interaction mechanisms among members of biocontrol flora is essential for understanding and constructing effective biocontrol communities to prevent and control cucumber Fusarium wilt. Microbial interactions can enhance environmental adaptability and expand ecological niches through insurance and complementary effects, thereby stabilizing microbial ecological functions and adapting to environmental pressures (Wang et al., 2022). Developing universal and stable biocontrol consortia represents a key strategy for improving biocontrol agents against cucumber Fusarium wilt.

In the initial phase of this study, *Glutamicibacter soli* JF_198 was confirmed to suppress disease by enhancing the soil microbial community. To investigate its mechanism of action, experiments using sterilized and non-sterilized rhizosphere soil with or without JF_198 inoculation revealed that disease suppression was closely associated with JF_198-induced alterations in the indigenous microbial community. Further analysis identified *Paenibacillus*-assigned OTU1077 as a candidate treatment-associated taxon potentially linked to disease suppression. Subsequent experiments further evaluated the growth response, competitive interaction, root colonization, and biocontrol potential of the cultivable isolate *Paenibacillus* sp. CJ136 under controlled conditions. This study provides preliminary insights into microbiome-associated disease suppression and may inform future development of microbial strategies for plant disease control.

## 2 Methods and materials

### 2.1 Soil, cucumber plants, and *F. Oxysporum* f. sp. *cucumerinum* inoculation

The soil used in this study was collected from the top 20 cm of an agricultural plot located in Hongqi District, Xinxiang City, Henan Province, China (35°30′N, 113°86′E). Historically, this land has been consistently cultivated with corn. This soil had no history of cucumber cultivation, a deliberate choice to minimize potential interference from pre-existing *F. oxysporum* f. sp. *cucumerinum* in the experimental system.

Cucumber seeds were surface-sterilized with 70% ethanol for 10 s, followed by disinfection with 3% sodium hypochlorite solution for 15 min. Seeds were thoroughly rinsed with sterile water. The seeds were allowed to germinate on sterile cotton gauze at 28 °C. Sterilized soil was validated by plating serial soil dilutions on nutrient agar plates and incubating them at 28 °C for 48 h. No visible microbial colonies were observed, confirming the effectiveness of soil sterilization. Plants were cultivated in greenhouses under controlled conditions: a light/dark cycle of 16/8 h, an average day-night temperature of 28/22 °C, and a relative humidity of 60%.

*F. oxysporum* f. sp. *cucumerinum* was cultured on PDA agar. The conidial suspension of *F. oxysporum* f. sp. *cucumerinum* was prepared following previously established protocols (Li et al., 2019; Li et al., 2023).

### 2.2 Pot experiment to test the disease resistance of cucumber plants in sterile and natural soils

The pot experiment included four treatment groups: cucumbers grown in natural soil (1) or sterile soil (2), and cucumbers grown in natural soil (3) or sterile soil (4) inoculated with strain JF_198. Each treatment contained three biological replicates, with 20 plants per replicate, resulting in 60 plants per treatment. The same replication design was used for disease severity assessment. As previously mentioned, these plants are maintained in the GnotoPot system (Kremer et al., 2021). *Fusarium oxysporum* f. sp. *cucumerinum* was cultured on PDA plates at 28 °C. Conidia were collected by rinsing the colony surface with sterile water and filtering through sterile gauze to remove mycelial fragments. The conidial concentration was determined using a hemocytometer and adjusted to the required concentration with sterile water. For pathogen inoculation, cucumber seedlings were inoculated by root drenching with the conidial suspension, with 20 mL of conidial suspension being applied to each pot. The inoculum was applied at the two-leaf stage and at the same time for all treatments, and the conidial suspension was gently mixed during inoculation to maintain uniformity. The final pathogen density was approximately 1.0 × 10^5^ conidia g^−1^ soil. Control treatments received the same volume of sterile water where appropriate. After inoculation, all plants were maintained under identical greenhouse conditions. Disease severity was assessed based on the percentage of yellowing/wilting leaves. Rhizosphere collection was performed by carefully removing plants from the pot and shaking to remove loose soil adhering to the roots. Subsequently, rhizosphere soil was collected using a sterile brush, which was considered the rhizosphere sample.

### 2.3 Rhizosphere transplant experiment

For the rhizosphere transplantation experiment, sterile soil was amended with 6% (w/w) rhizosphere inoculum derived from donor plant roots. Two treatments were established: (1) cucumbers grown in soil containing sterile soil and control group rhizosphere soil samples and (2) cucumbers grown in soil consisting of sterile soil mixed with JF_198 group rhizosphere soil samples. Each treatment contained three biological replicates, with 20 plants per replicate, resulting in 60 plants per treatment. Disease severity was assessed using the same replication design. Fusarium wilt severity was assessed based on the percentage of leaf yellowing/wilting. Additionally, we quantitatively assessed the severity of *F. oxysporum* f. sp. *cucumerinum* infection.

### 2.4 Amplicon sequencing and bioinformatics analysis

Bacterial 16S rRNA gene V3-V4 regions were amplified using 314F/806R primers. PCR products were sequenced using the Illumina NovaSeq platform. For microbiome sequencing, each treatment contained three biological replicates. In the original analysis, an OTU-based pipeline implemented in QIIME1 was used to filter, merge, and process the raw reads to obtain high-quality sequences for subsequent analysis. High-quality sequences were clustered into OTUs, and taxonomic classification was performed against the SILVA database. To address the methodological limitation of OTU-based clustering and to evaluate the robustness of the main microbiome patterns, the raw paired-end amplicon reads were additionally reprocessed using QIIME2 with the DADA2 plugin. Quality filtering, denoising, paired-end read merging, chimera removal, and amplicon sequence variant (ASV) inference were performed in the DADA2 workflow. Representative ASV sequences were taxonomically assigned using the SILVA ACT/SINA classifier. The ASV-based results were used as supplementary evidence to assess the consistency of the main OTU-based microbial community patterns. Principal coordinate analysis (PCoA) was performed based on Bray–Curtis dissimilarity using R 4.0. For the ASV-based supplementary analysis, differences in overall bacterial community structure between treatments were evaluated using PERMANOVA based on Bray–Curtis dissimilarity. For taxa-level comparisons involving multiple genera and OTUs, *p* values were adjusted using the Benjamini–Hochberg false discovery rate (FDR) procedure. Predefined pairwise comparisons, including alpha diversity indices and selected candidate taxa, were evaluated using Student’s *t*-test where appropriate. Relative abundance-based results were interpreted cautiously because of the compositional nature of amplicon sequencing data.

### 2.5 Isolation and identification of culturable bacteria

Culturable bacteria were isolated from the rhizosphere of cucumber plants cultivated. The isolation process used 1/10 tryptic soy agar medium, and bacterial identification was performed through 16S rRNA gene sequencing and analysis. Subsequently, the *in vitro* antagonistic activities of each *Paenibacillus* sp. isolate against *F. oxysporum* f. sp. *cucumerinum* were evaluated on PDA medium.

The inhibition rate was calculated as (D − B)/D × 100% (where D: colony diameter of the control group (CK); B: colony width of the pathogen in the treatment group). Each treatment was replicated three times, with only the pathogen cake inoculated as the control group (CK), and the results were expressed as the average of the three replicates.

Viability of *F. oxysporum* f. sp. *cucumerinum* was assessed using the FDA/PI staining technique, following established methods (Sun et al., 2024). A mycelial plug of *F. oxysporum* f. sp. *cucumerinum*, cultivated at 28 °C for 5 days, was introduced into PDB liquid medium. The cultures were incubated at 28 °C for 48 h. Subsequently, *Paenibacillus* sp. suspensions (with equal volumes of ddH_2_O added as the control) were introduced and cultured for an additional 12 h. A small quantity of newly formed *F. oxysporum* f. sp. *cucumerinum* hyphae was carefully extracted using tweezers and placed on a slide containing equal volumes of 50 mg L^−1^ FDA and 20 mg L^−1^ PI solution. The slide was maintained in darkness at 25 °C for 10 min. Hyphal staining was then observed using a fluorescence microscope.

Pot experiments were conducted to evaluate the biocontrol efficacy of this method on cucumber Fusarium wilt. The experiments involved three treatments: (1) inoculation with *F. oxysporum* f. sp. *cucumerinum* alone, (2) inoculation with *F. oxysporum* f. sp. *cucumerinum* and CJ136, and (3) inoculation with *F. oxysporum* f. sp. *cucumerinum*, CJ136, and JF_198. Each treatment contained three biological replicates, with 20 plants per replicate, resulting in 60 plants per treatment. Disease severity was assessed using the same replication design. Bacterial suspensions were applied through soil drenching to reach a final concentration of 1.0 × 10^5^ CFUg^−1^. The bacterial isolates were mixed in a 1:1 ratio for the combined treatments.

### 2.6 Bacterial growth curves

*G. soli* JF_198 and *Paenibacillus* sp. CJ136 were cultured in Murashige & Skoog (MS) minimal medium containing 2% root exudates of cucumber from JF_198-treated plants at 28 °C (Lee et al., 2024). The collection of cucumber root exudates was performed according to Kimani et al. (2016), with minor modifications. Briefly: the roots were gently rinsed with sterile double-distilled water (sterile ddH₂O) to avoid interference from MS medium components; subsequently, each plant was placed in a 100 mL Erlenmeyer flask, and the roots were submerged in 80 mL sterile ddH₂O for the collection of root exudates (Kimani et al., 2016). MS medium containing 2% root exudates of cucumber in the control group served as a control. Bacterial growth was assayed using a microplate reader at an Optical Density (OD) 600 for 24 h. Each treatment was performed with three biological replicates.

### 2.7 Competition assay

The competition between *G. soli* JF_198 and *Paenibacillus* sp. CJ136 (spontaneous streptomycin-resistant mutant) for nutrients was investigated as follows. Bacteria suspensions were adjusted to OD_600_ = 0.2 in MS medium and mixed at a 1:1 ratio. Root exudates from different treatments were added to the mixture at 2% specified concentrations and incubated at 28 °C. Colony-forming units (CFUs) of both strains were determined using the dilution plate method on LB and LB with 50 μg mL^−1^ tetracycline media at 24 h after co-incubation started. Each treatment was performed with three biological replicates.

### 2.8 Colonization assay

The plasmids pHY300PLK and pUC57-GFPMUT3 were enzymatically digested by restriction endonuclease, and the pHY300 plasmid was ligated with the GFPMUT3 fragment. After heat shock transformation, the plasmid was introduced into *Escherichia coli* DH5α to construct the fluorescent protein expression vector pPPGFP. The constructed fluorescent protein expression vector pPPGFP was transformed via electroporation to generate the green fluorescent protein (GFP)-tagged strains *Paenibacillus* sp. CJ136 (Supplementary Figure S1). To assess the impact of JF_198 on the root colonization capability of *Paenibacillus* sp. CJ136, root colonization assays were performed following the protocol described by Dragoš et al. (2018). The surface fluorescence of roots was examined using a fluorescence microscope (Ni-U/Ni-E fluorescence microscope, NIKON, Japan), and the fluorescence intensity was quantified using ImageJ software (Sun et al., 2022). Each treatment was performed with three biological replicates.

### 2.9 Statistical analysis

Statistical analyses were conducted using R version 4.0.3 or GraphPad Prism 8 software. Figures were created using the ggplot2 R package or GraphPad Prism 8. Comprehensive statistical analyses are detailed in the respective figure legends.

## 3 Results

### 3.1 *G. soli* JF_198 alters the cucumber rhizosphere microbiome and suppresses Fusarium wilt disease

**Figure 1:**
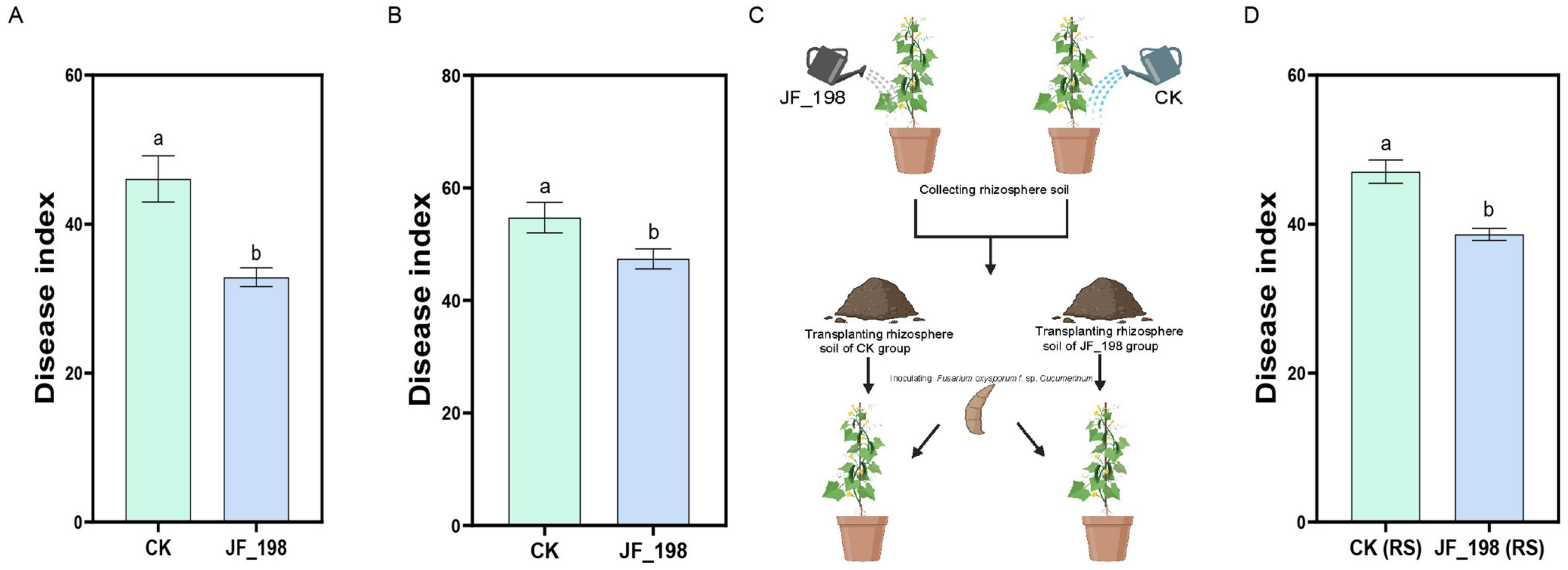
Strain JF_198-induced cucumber rhizosphere microbiota recruitment and Fusarium wilt disease resistance. **(A)** Fusarium wilt disease severity of cucumber plants grown in natural soil. **(B)** Fusarium wilt disease severity of cucumber plants grown in sterile soil. **(C)** Schematic diagram of the rhizosphere transplantation experiment used to evaluate the contribution of JF_198-associated rhizosphere microbiota. **(D)** Disease severity of cucumber plants after rhizosphere microbiota transplantation. Cucumber seedlings were cultivated in soils without JF_198 amendment (CK) or with JF_198 amendment (JF_198). Data are shown as mean ± SD. Each treatment contained three biological replicates, with 20 plants per replicate. Different lowercase letters above the bars indicate significant differences according to Duncan’s multiple range test (*p* < 0.05). Exact *p* values, sample sizes, and effect sizes are provided in Supplementary Table S8. Panel C was created with BioRender.com.

We screened 42 bacteria to obtain the target strains that can reduce the incidence index of cucumber Fusarium wilt. Compared to the uninoculated control, cucumber plants that were inoculated with *F. oxysporum* f. sp. *Cucumerinum* and treated with JF_198 showed a reduced disease incidence index of cucumber Fusarium wilt, decreased by 28.6% (Figure 1A). Notably, JF_198 exhibited no direct antagonistic activity on *F. oxysporum* f. sp. *cucumerinum* (Supplementary Figure S2A), suggesting that JF_198 may exert its influence by modulating soil microbial communities. To investigate this hypothesis, we examined the effect of strain JF_198 on cucumber Fusarium wilt resistance in sterile soil. The results indicated that while the disease incidence of cucumber Fusarium wilt decreased by 13.4% in sterile soil, the incidence index decreased by 28.6% in natural soil conditions when treated with strain JF_198 (Figure 1B). These results indicate that JF_198 enhances Fusarium wilt resistance partly by modulating the soil microbiome. To further explore this, we collected the rhizosphere soil microbial community from natural soil and introduced it to sterile soil (Figure 1C). We observed that the rhizosphere microorganisms treated with JF_198 demonstrated enhanced disease resistance (Figure 1D). In conclusion, strain JF_198 appears to stimulate plant disease resistance by influencing the rhizosphere microorganisms of cucumber. PCR amplification using the universal 16S rRNA primer 27F/1492R yielded a 1,418-bp band. Homology alignment analysis revealed that the 16S rRNA sequence of strain JF_198 exhibited a high similarity of over 98% with the 16S rRNA sequence of *G. soli* strain SYB2 (GenBank accession number NR 044338.1) (Supplementary Figure S3). Phylogenetic analysis revealed that strain JF_198 clustered with *G. soli* strain SYB2 into the same branch (Supplementary Figure S2B).

### 3.2 Changes in rhizosphere microbial community

**Figure 2:**
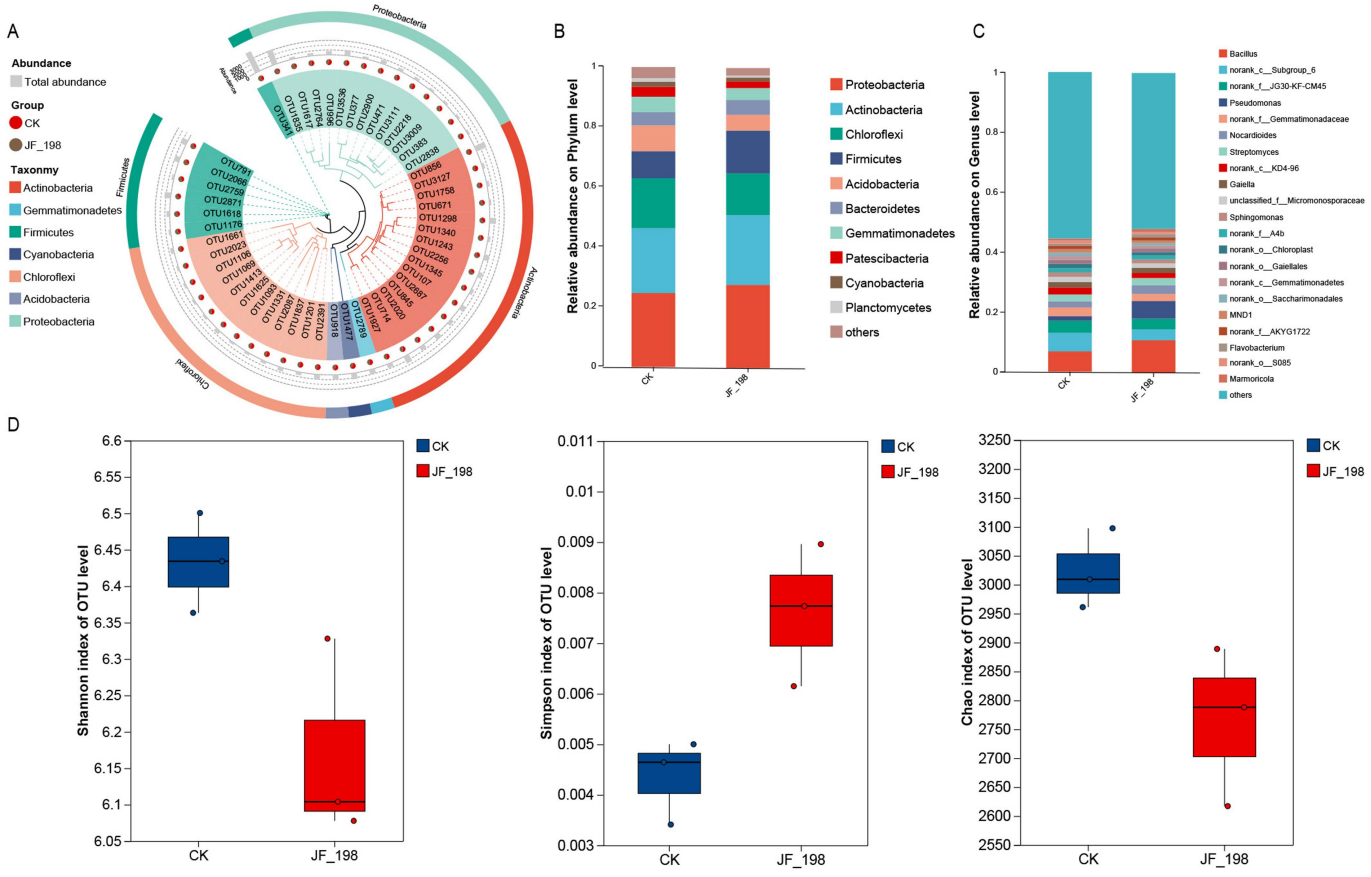
Composition and diversity of bacterial communities in cucumber rhizosphere soil. **(A)** Phylogenetic distribution of representative bacterial OTUs and the relative abundance of dominant phyla across soil samples. **(B)** Bacterial community composition at the phylum level. **(C)** Bacterial community composition at the genus level. **(D)** Shannon, Simpson, and Chao1 indices of bacterial alpha diversity in rhizosphere soil. For microbiome sequencing, each treatment contained three biological replicates. Alpha diversity indices showed treatment-associated trends based on predefined pairwise comparisons, but these differences did not remain significant after Benjamini–Hochberg FDR correction. Exact *p* values, FDR-adjusted *p* values, sample sizes, and effect sizes are provided in Supplementary Table S8.

Firstly, a total of 3,438 operational taxonomic units (OTUs) were obtained from the 16S sequencing results (Supplementary Figure S4). Taxonomic analysis revealed that the cucumber rhizosphere bacterial community was composed of seven dominant phyla (Figure 2A), with Proteobacteria (26.17%) being the most abundant phylum, followed by Actinobacteria (22.43%), Chloroflexi (15.32%), Firmicutes (11.65%), Acidobacteria (6.92%), Bacteroidetes (4.67%), and Gemmatimonadetes (4.58%) (Figure 2B). Compared to the control (CK), Patescibacteria, Elusimicrobia, and Lentisphaerae showed decreasing trends in the JF_198 treatment group. Furthermore, Figure 2C illustrates the relative abundance of the top 18 most abundant genera (>1% relative abundance), with the dominant genera including *Bacillus*, norank_c_Subgroup-6, Norank_f_JG30-KF-CM45, *Pseudomonas*, norank_f_Gemmatimonadaceae, *Nocardioides*, and *Streptomyces*. In comparison with CK, several genera showed treatment-associated changes in relative abundance in the JF_198 treatment group (Supplementary Table S2). In particular, *Pseudomonas* (5.89%) and *Paenibacillus* (0.79%) showed increased relative abundance in the JF_198 treatment group (Supplementary Table S2). After Benjamini–Hochberg FDR correction, these genus-level changes were interpreted cautiously as treatment-associated enrichment trends rather than definitive statistically significant differences. Of particular note, the abundance of *Paenibacillus* nearly doubled in the JF_198 treatment group. Figure 2D shows the alpha diversity indices of the rhizosphere bacterial communities. Compared with CK, the Shannon and Chao1 indices showed decreasing trends in the JF_198 treatment group, whereas the Simpson index showed an increasing trend based on predefined pairwise comparisons. However, these alpha diversity changes did not remain significant after Benjamini–Hochberg FDR correction (Figure 2D; Supplementary Table S8). Principal coordinate analysis (PCoA) based on Bray–Curtis dissimilarity showed a tendency toward separation between CK and JF_198-treated samples at the OTU level; however, the difference was not statistically significant (*R* = 0.5185, *p* = 0.0980; Supplementary Figure S5).

To address the methodological limitation of the original OTU-based analysis, we further reprocessed the raw amplicon reads using a QIIME2/DADA2-based ASV workflow. The ASV-based reanalysis generated 1,845 ASVs, and the DADA2 denoising statistics are provided in Supplementary Table S4. At the phylum level, the ASV-based community composition showed dominant bacterial groups broadly comparable to those observed in the OTU-based analysis (Supplementary Figure S6A). Bray–Curtis PCoA based on ASV profiles showed limited separation between CK and JF_198 samples; however, this difference was not statistically significant by PERMANOVA (*R*^2^ = 0.200, *p* = 1.000; Supplementary Figure S6B). Nevertheless, *Paenibacillus*-related ASVs showed higher relative abundance in the JF_198 treatment than in CK, with the group mean increasing from approximately 0.10% in CK to 0.60% in the JF_198 treatment (Supplementary Figure S6C and Supplementary Table S5). A comparison between the OTU-based and ASV-based analyses further showed that the enrichment trend of *Paenibacillus*-related taxa under JF_198 treatment was consistent between the two approaches (Supplementary Table S6). These ASV-based results were provided as supplementary evidence supporting the consistency of the main OTU-based enrichment trend.

### 3.3 Responsive microbial taxa and isolation of *Paenibacillus* sp.

**Figure 3:**
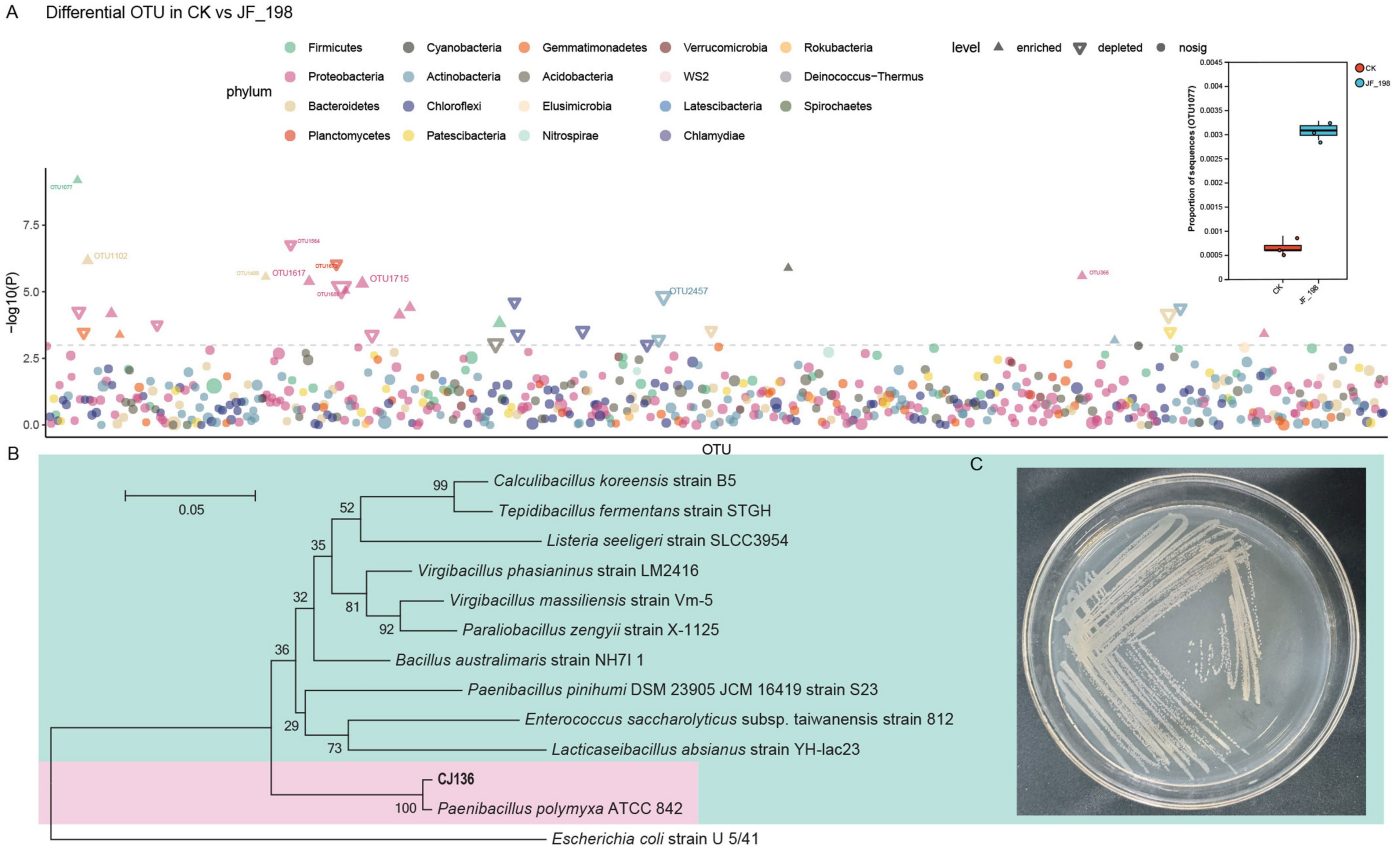
Treatment-associated OTU trends and isolation of candidate *Paenibacillus* sp. CJ136. **(A)** OTU-level relative abundance trends between CK and JF_198 treatments. OTU1077, assigned to *Paenibacillus* sp., showed increased relative abundance in JF_198-treated samples and was selected as a candidate taxon for subsequent culture-based isolation and functional validation. The inset plot displays the relative abundance of OTU1077. For OTU-level relative abundance analysis, each treatment contained three biological replicates. Exact *p* value, FDR-adjusted *p* value, sample size, and effect size are provided in Supplementary Table S8. **(B)** Phylogenetic tree based on 16S rRNA gene sequences showing the relationship between CJ136 and closely related bacterial strains. **(C)** Colony morphology of *Paenibacillus* sp. CJ136 on PDA plates.

We further investigated the association between specific bacterial OTUs and JF_198 treatments, as well as their relationship with cucumber Fusarium wilt severity. OTU-level relative abundance analysis identified 138 bacterial OTUs showing treatment-associated variation under JF_198 treatment (Supplementary Table S3). Among these OTUs, 30 showed more than a two-fold increase in relative abundance in the JF_198 treatment compared with CK. Among the treatment-associated OTUs, OTU1077, annotated as *Paenibacillus* sp., showed a marked enrichment trend in the JF_198 treatment and was therefore selected as a candidate taxon for subsequent culture-based isolation and functional validation. The relative abundance of *Paenibacillus*-assigned OTU1077 increased from 0.065% in CK to 0.305% in the JF_198 treatment (Figure 3A; Supplementary Table S3). Moreover, the relative abundance of *Paenibacillus*-assigned OTU1077 was negatively correlated with the cucumber Fusarium wilt severity index (*R* = −0.95, *p* = 0.0036; Supplementary Figure S7). Based on these observations, we hypothesized that the suppressive effect of JF_198 against cucumber Fusarium wilt may involve interactions with *Paenibacillus* spp. To investigate this, we isolated cultivable Bacillales from the rhizosphere soil microbial community. In total, 76 Bacillales isolates were isolated, screened, and identified from the rhizosphere soil microbial communities of the CK and JF_198 treatments. These isolates represented 17 phylogenetically distinct groups based on 16S rRNA gene sequences. From these 76 Bacillales isolates, we selected *Paenibacillus* sp. CJ136, which shared >96% 16S rRNA gene sequence identity with OTU1077 (Supplementary Figure S8). Further phylogenetic analysis of the 16S rRNA sequence of *Paenibacillus* sp. CJ136 showed that CJ136 clustered closely with *Paenibacillus polymyxa* (Figure 3B). Morphologically, the colonies of *Paenibacillus* sp. CJ136 were white and circular, with a smooth, slightly convex surface and sticky texture (Figure 3C).

### 3.4 Antifungal properties of *Paenibacillus* sp. CJ136 against *F. oxysporum* f. sp. *cucumerinum*

**Figure 4:**
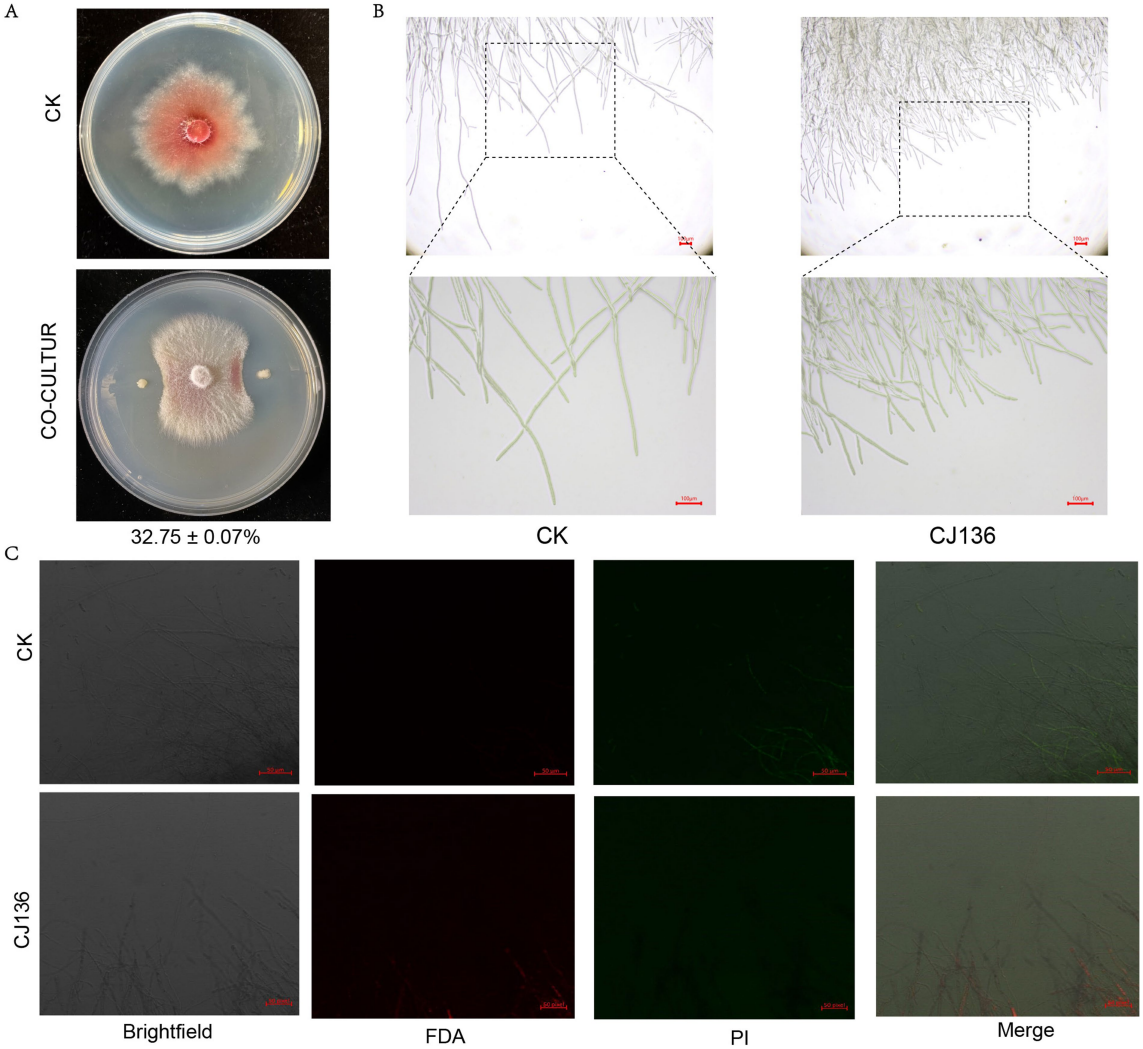
Isolated Bacillales and the effects of CJ136 on cucumber seedling disease resistance. **(A)** Cladogram illustrating the phylogenetic relationships among 76 rhizosphere soil Bacillales and OTU1077. Leaf labels indicate representative species-level taxonomy. The inner ring represents the genus-level taxonomy, while the outer ring denotes the soil from which the strain was isolated. **(B)** Antagonistic effect of JF_198 strain against *F. oxysporum* f. sp. *cucumerinum* and optical microscope analysis. **(C)** Microscopic observation of mycelia stained with fluorescein diacetate and propidium iodide.

Our findings revealed that *Paenibacillus* sp. CJ136 demonstrated *in vitro* antagonistic activity against *F. oxysporum* f. sp. *cucumerinum* on potato dextrose agar (PDA) medium (Figure 4A). Compared to the control (without CJ136 inoculation), PDA plates inoculated with CJ136 exhibited a significant antifungal zone (inhibition rate of 32.75%), indicating that CJ136 substantially inhibited the hyphal growth of *F. oxysporum* f. sp. *cucumerinum*. Examination of the hyphal morphology at the edge of the *Paenibacillus* sp. CJ136 colony showed severe morphological distortions in the closest hyphae, while the control group exhibited normal hyphal morphology (Figure 4B). FDA/PI dual staining validated the hyphal morphological changes. Control hyphae exhibited regular morphology with green fluorescence, whereas CJ136-treated hyphae displayed abnormal morphology and significantly enhanced red fluorescence. Conversely, the CJ136 treatment group displayed abnormal hyphal morphology and an inverse staining pattern (Figure 4C). These results consistently indicate that the CJ136 strain exhibits potent inhibitory efficiency against *F. oxysporum* f. sp. *cucumerinum* by disrupting hyphal membrane integrity.

### 3.5 Interactions between *Paenibacillus* sp. CJ136 and *G. soli* JF_198 under root-exudate conditions

**Figure 5:**
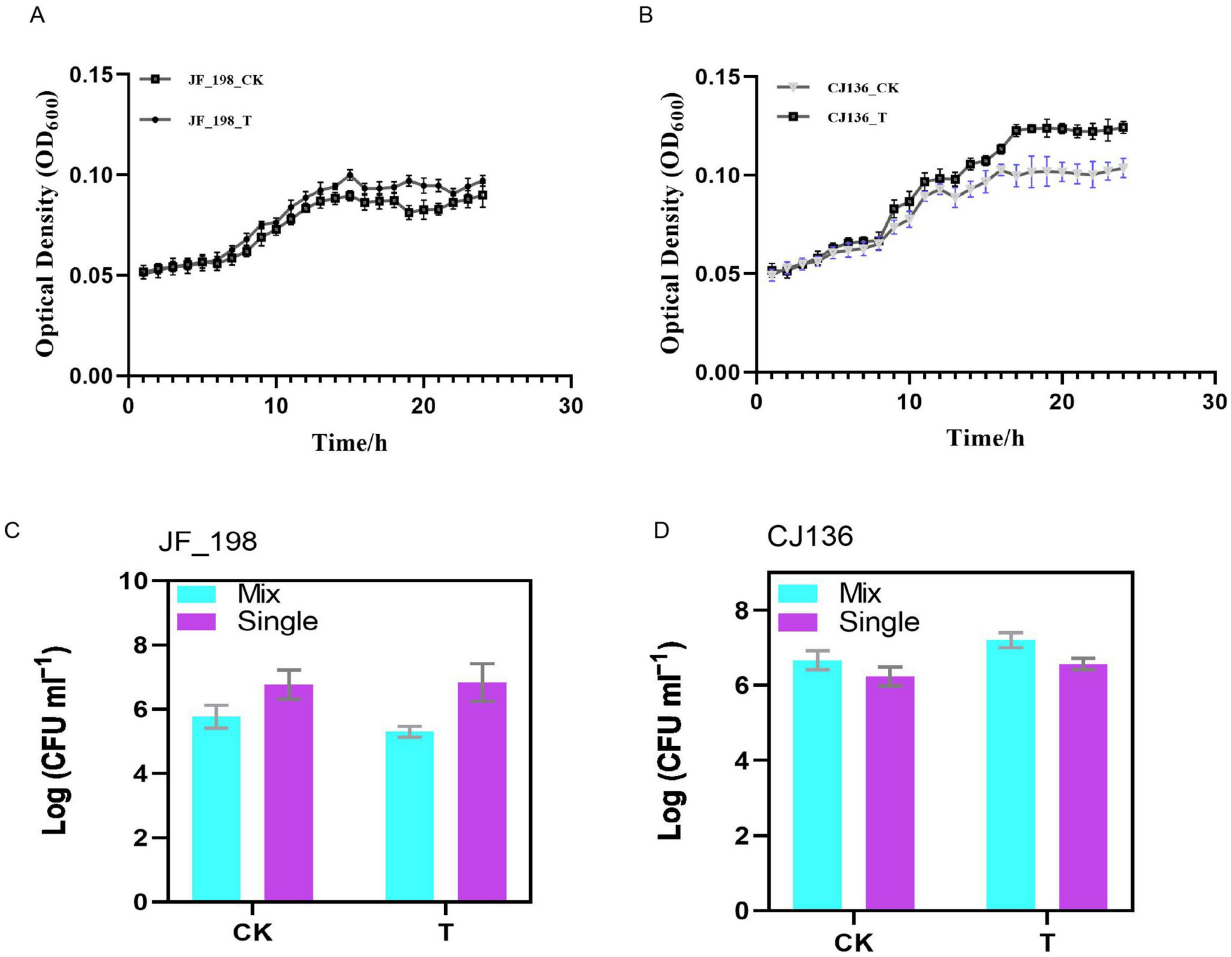
Root-exudate-associated growth responses and competitive interaction between *Paenibacillus* sp. CJ136 and *G. soli* JF_198. **(A, B)** Growth curves of *Paenibacillus* sp. CJ136 and *G. soli* JF_198 in Murashige and Skoog (MS) medium supplemented with 2% cucumber root exudates collected from control plants (CK) or JF_198-treated plants (T). OD600 was measured every hour for 24 h. **(C, D)** Viable cell numbers of CJ136 and JF_198 after 24 h under single-culture or mixed-culture conditions with cucumber root exudates. Data are shown as mean ± SD from three biological replicates. Exact *p* values, sample sizes, and effect sizes are provided in Supplementary Table S8.

To characterize the interaction between *Paenibacillus* sp. CJ136 and *G. soli* JF_198, the growth of both strains was assessed in MS medium supplemented with 2% (v/v) cucumber root exudates. *Paenibacillus* sp. CJ136 exhibited enhanced growth in MS medium supplemented with root exudates collected from JF_198-treated cucumber plants compared with the control root-exudate treatment. Meanwhile, *Paenibacillus* sp. CJ136 exhibited faster growth kinetics compared to *G. soli* JF_198 (Figures 5A,B). When *Paenibacillus* sp. CJ136 and *G. soli* JF_198 were co-cultured in MS medium supplemented with 2% root exudates, the growth of *G. soli* JF_198 was significantly inhibited by *Paenibacillus* sp. CJ136 (*p* < 0.05) (Figures 5C,D). These results indicate that JF_198-treated root exudates favored the growth of CJ136 under the tested conditions, whereas direct co-culture showed a competitive interaction in which CJ136 inhibited the growth of JF_198.

### 3.6 *G. soli* JF_198 and *Paenibacillus* sp. CJ136 enhance cucumber defense against Fusarium wilt

**Figure 6:**
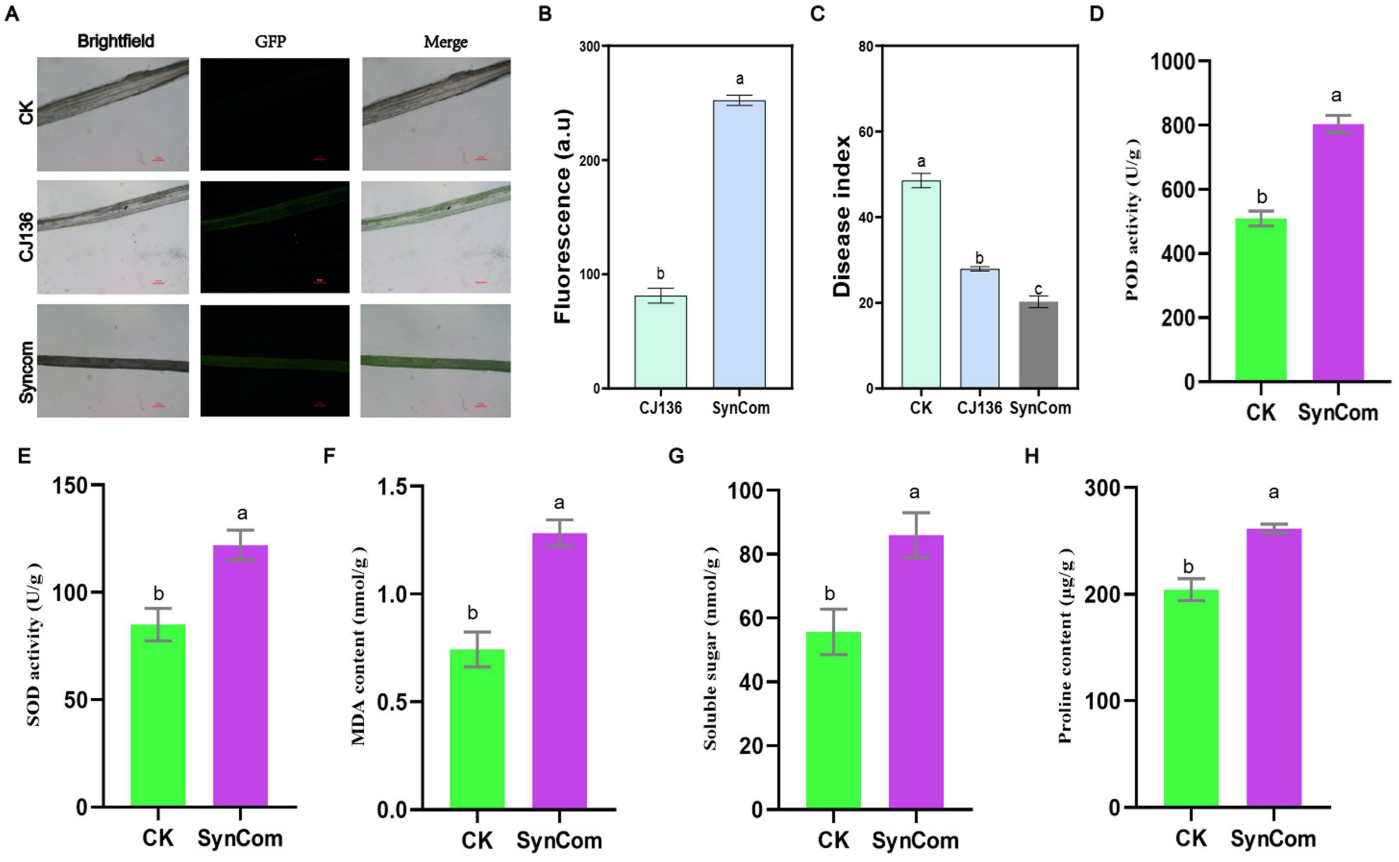
Effects of CJ136 and SynCom on cucumber *Fusarium* wilt suppression and root colonization. **(A)** Root colonization of GFP-tagged CJ136 under CK, CJ136, and SynCom treatments. **(B)** Quantitative analysis of CJ136 colonization on cucumber roots. **(C)** Disease index of cucumber *Fusarium* wilt under different treatments. CK, pathogen-inoculated control; CJ136, treatment with *Paenibacillus* sp. CJ136; SynCom, combined treatment with *G. soli* JF_198 and *Paenibacillus* sp. CJ136. **(D–H)** POD activity, SOD activity, MDA content, soluble sugar content, and proline content in cucumber roots. Unless otherwise stated, data are shown as mean ± SD from three biological replicates. For pot experiments, each treatment contained three biological replicates, with 20 plants per replicate. Different lowercase letters above the bars indicate significant differences according to Duncan’s multiple range test (*p* < 0.05). Exact *p* values, sample sizes, and effect sizes are provided in Supplementary Table S8.

To assess JF_198’s impact on *Paenibacillus* sp. CJ136 root colonization, we performed surface colonization assays. Fluorescence microscopy demonstrated that co-inoculation with JF_198 and GFP-tagged CJ136 significantly enhanced GFP signal intensity on the root surface (Figures 6A,B). Furthermore, it was observed that *Paenibacillus* sp. CJ136 significantly reduced the disease index of cucumber Fusarium wilt. Remarkably, the combined application of *G. soli* JF_198 and *Paenibacillus* sp. CJ136 resulted in a substantial decrease in the incidence index of cucumber Fusarium wilt (Figure 6C). These findings suggest that *G. soli* JF_198 may enhance the proliferation of the *Paenibacillus* sp. CJ136 population and significantly lower the incidence index of cucumber Fusarium wilt. To characterize host physiological responses, we measured the activity of antioxidant enzymes POD and SOD, the content of MDA, and osmotic agents in cucumber (Figures 6D–H). The result showed that the activity of POD and SOD in cucumber inoculated with JF_198 and CJ136 was significantly elevated (Figures 6D,E). In addition, MDA content showed a significant treatment-associated increase under SynCom treatment compared with CK (Figure 6F). Consistent with the disease index of cucumber Fusarium wilt, the content of proline and soluble sugars was higher in cucumber inoculated with JF_198 and CJ136 than in the control, indicating higher disease resistance (Figures 6G,H). Collectively, these results suggest that the combined application of JF_198 and CJ136 could enhance cucumber defense responses against Fusarium wilt under controlled pot conditions.

## 4 Discussion

The intricate interactions between agricultural soil microorganisms and their host plants are well documented; in particular, their impacts on plant growth and disease resistance are a focus of considerable research. Studies demonstrate that host genotypes, in conjunction with environmental factors, influence the composition of indigenous microbiota (Jin et al., 2022; Wang et al., 2024). Nevertheless, the introduction of exotic species often results in significant disturbances to native microbial communities. This investigation centered on cross-species microbial interactions, demonstrating that changes in the rhizosphere soil community induced by specific microbial taxa play a crucial role in enhancing plant disease resistance, based on the concept of biocontrol (Sun et al., 2022; Tao et al., 2020).

Recent studies have highlighted the significant potential of *Glutamicibacter* sp. in promoting plant growth and enhancing plant resilience to biotic and abiotic stresses (Lata et al., 2024; Petrović et al., 2023). However, the role of *Glutamicibacter* sp. in controlling fungal soil-borne diseases remains poorly characterized. This study investigated the efficacy of *G. soli* JF_198 against cucumber Fusarium wilt. Interestingly, the results indicated that *G. soli* JF_198 effectively suppressed the disease, despite the fact that it did not directly antagonize the pathogen.

In the present study, the inhibitory effect of *G. soli* JF_198 on cucumber Fusarium wilt was associated with the enrichment of specific bacterial groups in the cucumber rhizosphere, particularly *Paenibacillus* bacteria. *Paenibacillus* bacteria are considered prime candidates for developing biological control agents, as their ability to form endospores significantly improves their persistence and colonization efficiency in the rhizosphere. Additionally, *Paenibacillus* sp. often possess nitrogen fixation, phosphate solubilization, and other plant growth-promoting abilities (Dobrzyński and Naziębło, 2024). Du et al. demonstrated that *Paenibacillus* sp. NSY50 mitigated *F. oxysporum* f. sp. *cucumerinum* stress by activating GSH metabolism and improving redox balance (Du et al., 2022). Notably, the application of JF_198 was associated with an enrichment trend of OTU1077, which was assigned to *Paenibacillus*. These findings suggest a potential ecological association between JF_198 treatment and *Paenibacillus*-related taxa in the cucumber rhizosphere. However, this association should not be interpreted as mutualism because direct co-culture assays indicated a competitive interaction between CJ136 and JF_198.

To investigate the potential role of *Paenibacillus*-related bacteria, we selected the cultivable isolate *Paenibacillus* sp. CJ136, which was related to OTU1077 based on 16S rRNA gene sequence similarity, for subsequent functional assays. This bacterium substantially inhibited the hyphal growth of *F. oxysporum* f. sp. *cucumerinum*. To elucidate this, culture experiments were designed to compare the growth trends of *G. soli* JF_198 and *Paenibacillus* sp. CJ136 in monoculture and coculture systems. Results indicated that co-culture promoted the growth of *Paenibacillus* sp. CJ136, while restricting the growth of *G. soli* JF_198, compared to their respective solo cultures. Microbial communities exhibit a diverse range of interactions, from mutualism to competition, which collectively shape dynamic microbiota. These interactions can involve cross-feeding for mutual benefit or adhere to competitive exclusion principles, potentially resulting in mutual disadvantage (Faust and Raes, 2012). These results suggest an asymmetric interaction under the tested conditions, in which CJ136 benefited under JF_198-associated root-exudate conditions, whereas JF_198 was inhibited during direct co-culture.

Based on the observed enrichment trend of *Paenibacillus*-related taxa and the enhanced root colonization of CJ136 in the presence of JF_198, we hypothesized that JF_198 might indirectly enhance the biocontrol contribution of CJ136 in the rhizosphere environment. This hypothesis was subsequently validated through potting experiments. The findings demonstrate that *G. soli* JF_198 facilitates the colonization of *Paenibacillus* sp. CJ136 in cucumber roots and enhances host plant resistance to cucumber Fusarium wilt. The capacity of beneficial bacteria to enhance plant resistance to specific pathogens has been well documented, with this resistance being induced by metabolites or volatile organic compounds (VOCs; Rani et al., 2023). For example, *Streptomyces* sp. strain HL-66 has been observed to induce systemic resistance in walnut by secreting the extracellular polysaccharide EPS66A, increasing the activities of enzymes associated with disease resistance (e.g., catalase, superoxide dismutase, and peroxidase), which in turn improves the ability of walnut plants to cope with bacterial blight (Wu et al., 2024). Consistent with this mechanism, pot experiments demonstrated that combining JF_198 with CJ136 achieves control of cucumber blight by enhancing cucumber plants’ disease resistance.

It should also be noted that the microbiome analysis was based on relative abundance data from three biological replicates per treatment. Although three biological replicates are commonly used in amplicon-based rhizosphere microbiome studies, this sample size may limit the statistical power to detect subtle community-level differences. In addition, because absolute quantification using qPCR or other methods was not performed, the observed increase in *Paenibacillus*-related taxa should be interpreted as a relative abundance-based enrichment trend rather than direct evidence of absolute population expansion. Therefore, the microbiome results were interpreted cautiously and were considered together with culture-based isolation and functional validation of CJ136. It should also be noted that all disease suppression and colonization experiments in this study were conducted under controlled pot conditions. Although these experiments provide useful evidence for the potential association between JF_198 treatment, *Paenibacillus* sp. CJ136, and cucumber Fusarium wilt suppression, the stability and efficacy of this microbial combination under field conditions remain to be validated. Soil heterogeneity, native microbial communities, environmental fluctuations, and crop management practices may influence the establishment and function of introduced microorganisms. Therefore, further field trials and multi-site validation will be needed before extending these findings to practical agricultural applications.

In conclusion, under controlled pot conditions, our findings indicate that a microbial consortium comprising *G. soli* JF_198 and *Paenibacillus* sp. CJ136 could enhance cucumber resistance to Fusarium wilt. Specifically, *G. soli* JF_198 treatment was associated with increased abundance and root colonization of *Paenibacillus*-related bacteria, including CJ136, which may contribute to enhanced disease suppression in the cucumber rhizosphere. However, because no metabolomic profiling, chemotaxis assay, or molecular validation was performed in the present study, the specific metabolites or molecular signals mediating this association remain to be identified. Further field trials and multi-site validation will also be required to evaluate the stability, efficacy, and practical applicability of this microbial combination under realistic agricultural conditions.

## Data Availability

The original contributions presented in the study are included in the article/Supplementary material, further inquiries can be directed to the corresponding authors.
