# Supplementary material for: Glutamicibacter soli JF_198 stimulates the rhizosphere colonization of indigenous Paenibacillus sp. to suppress cucumber Fusarium
Source: Front Microbiol. 2026 May 26;17:1843711. doi: 10.3389/fmicb.2026.1843711 (PMC13246374; doi:10.3389/fmicb.2026.1843711)
Supplement: Supplementary file 1 [file Data_Sheet_1.docx]

**SUPPORTING INFORMATION TO:**

***Glutamicibacter soli* JF_198 stimulates the rhizosphere colonization of indigenous *Paenibacillus* sp. to suppress cucumber Fusarium**

**Supplementary Figures**

**
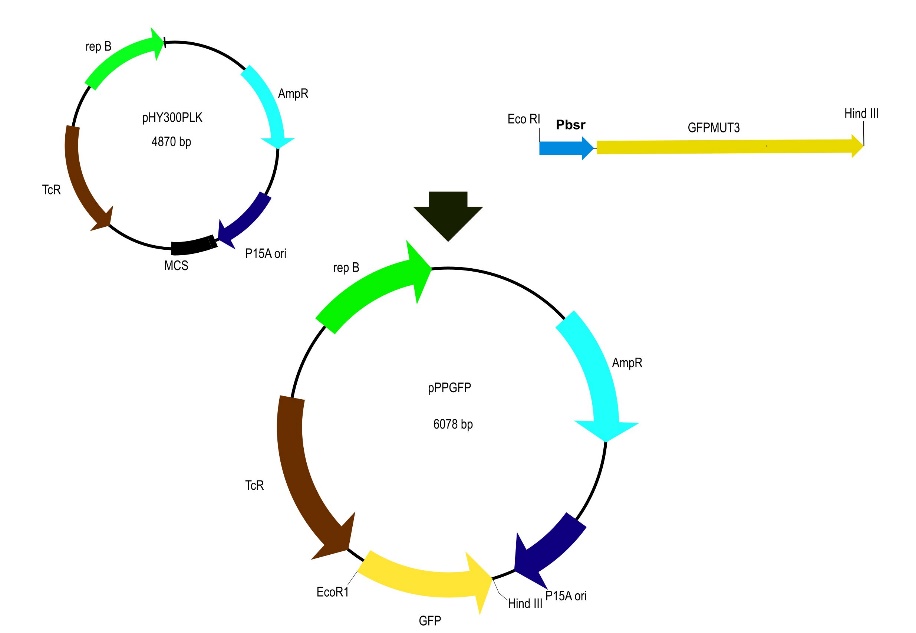
**

Figure S1 Construction of GFP expression vector of *Paenibacillus polymyxa* CJ136.


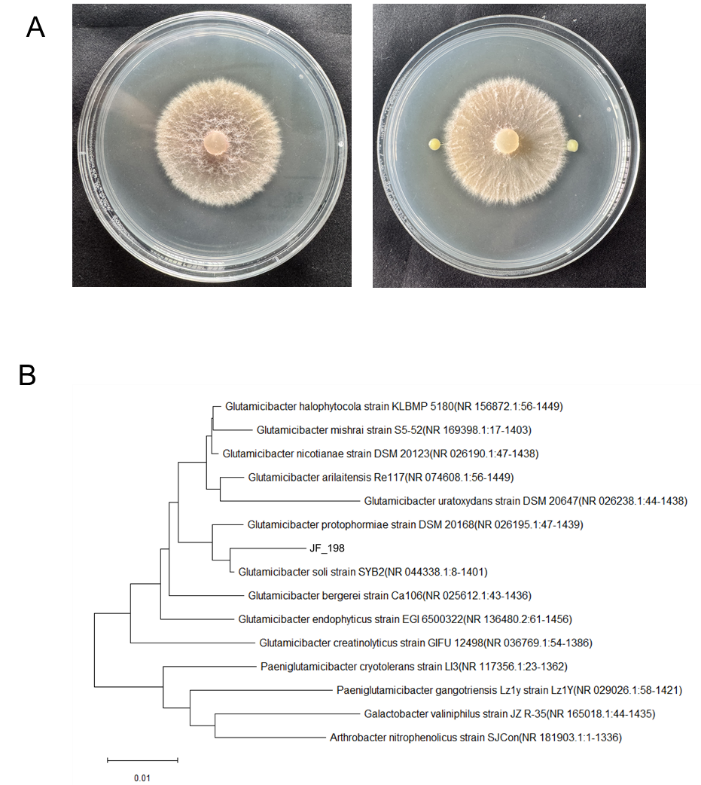


Figure S2 Inhibitory effect of JF_198 on the mycelium growth of phytopathogenic fungi (A) and the phylogenetic analysis based on 16 S rRNA (B).


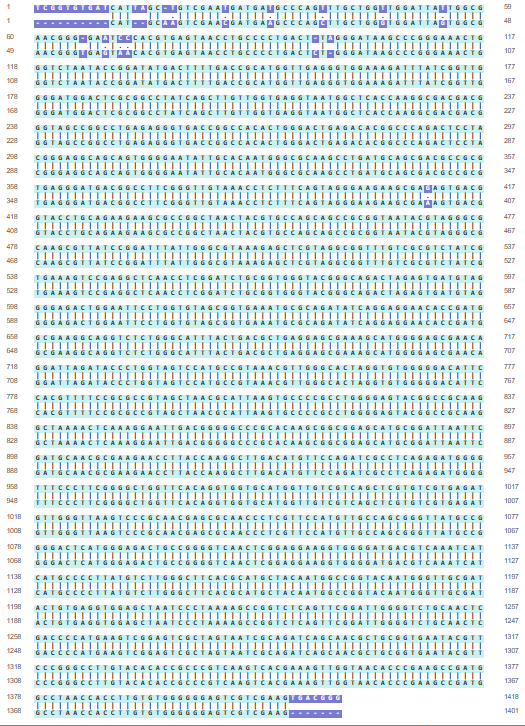


Figure S3 The 16S rRNA sequence similarity analysis of strain JF_198 and *G. soli* strain SYB2.


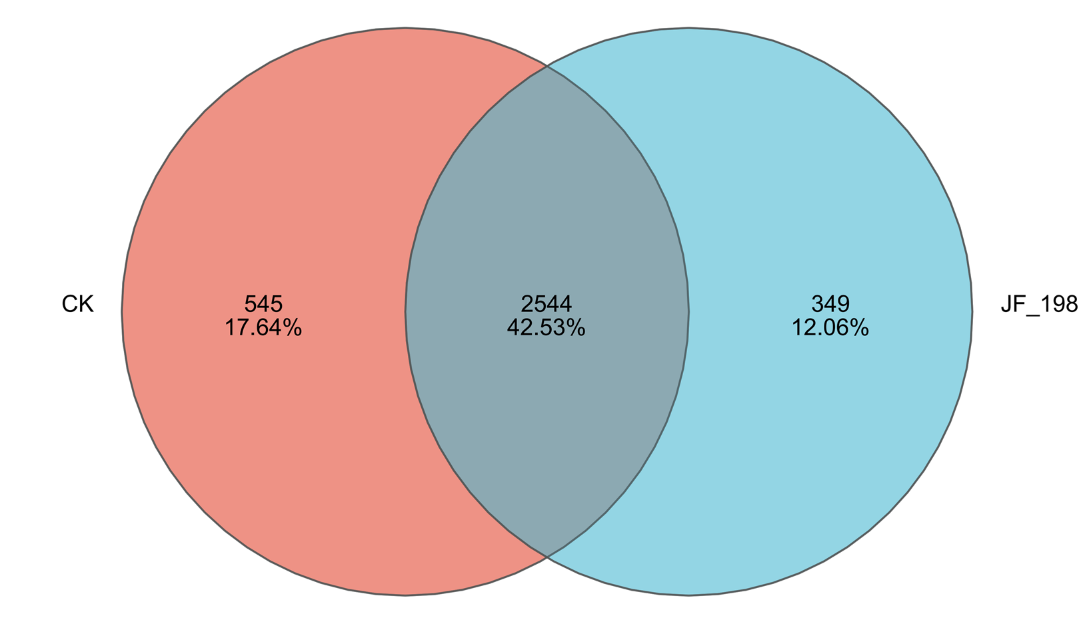


Figure S4 Analysis of OTU level Venn diagram based on cucumber rhizosphere microbial community.


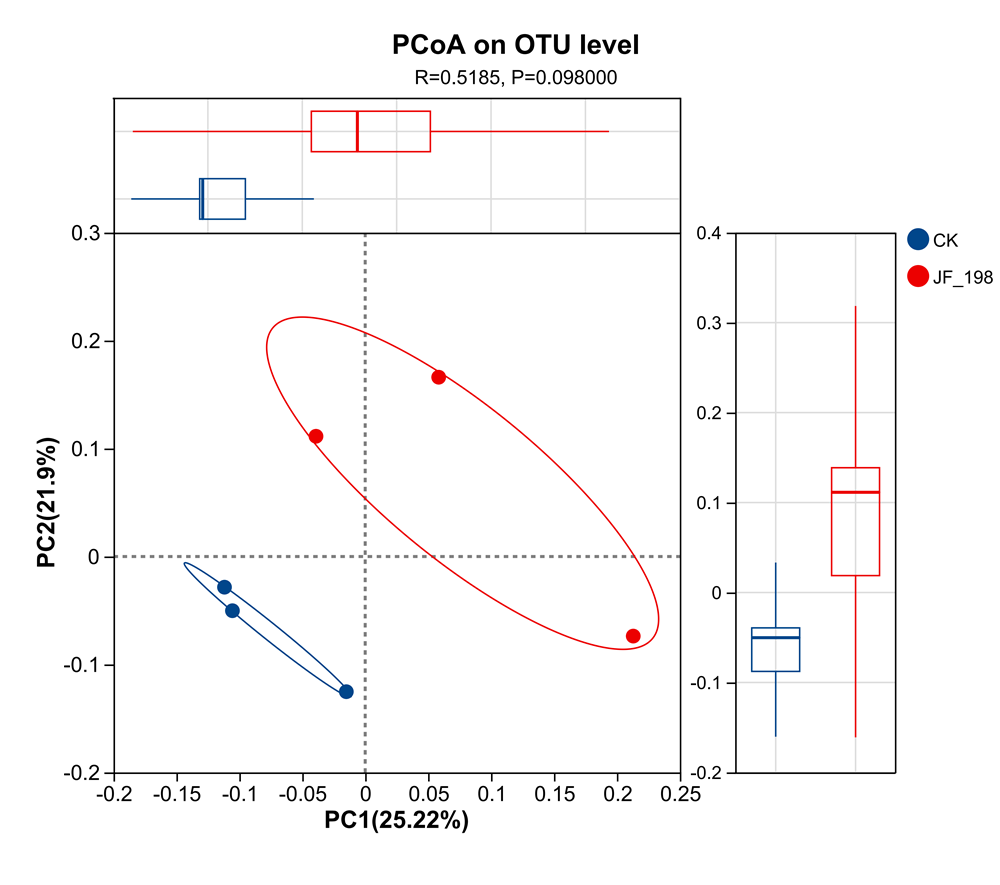


Figure S5 Principal coordinate analysis (PCoA) for bacterial communities.


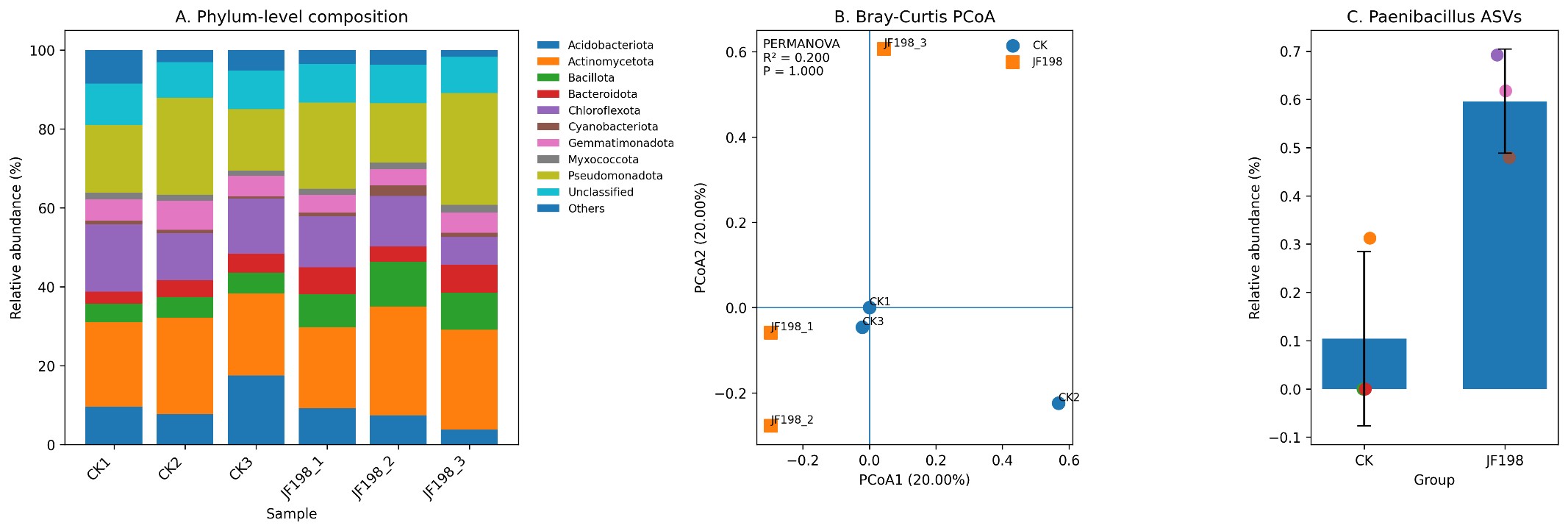


**Figure S6. ASV-based reanalysis of cucumber rhizosphere bacterial communities using QIIME2/DADA2.**
(A) Phylum-level community composition based on the ASV table.
(B) Principal coordinate analysis (PCoA) based on Bray–Curtis dissimilarity of ASV profiles. PERMANOVA was used to test group differences between CK and JF_198 treatments.
(C) Relative abundance of *Paenibacillus*-related ASVs in CK and JF_198-treated samples. Bars indicate group means, and points indicate individual biological replicates.


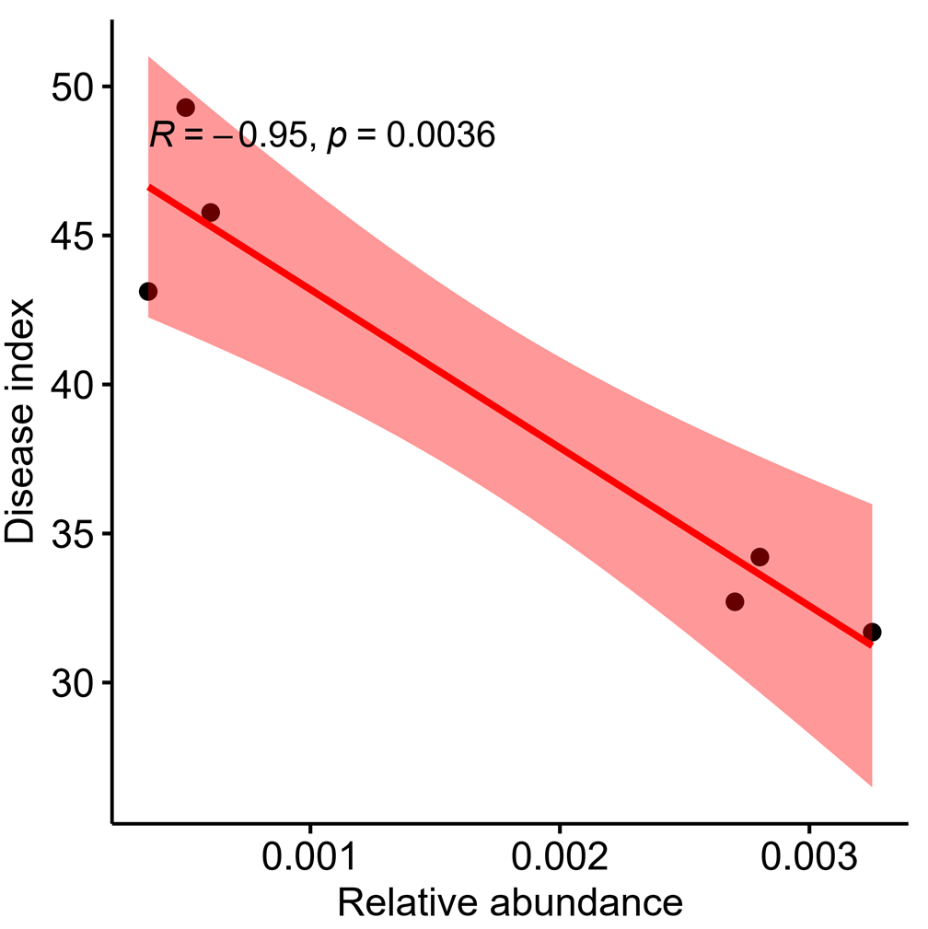


Figure S7 Scatter plots illustrating the correlation between disease index and the relative abundance of the OTU1077.


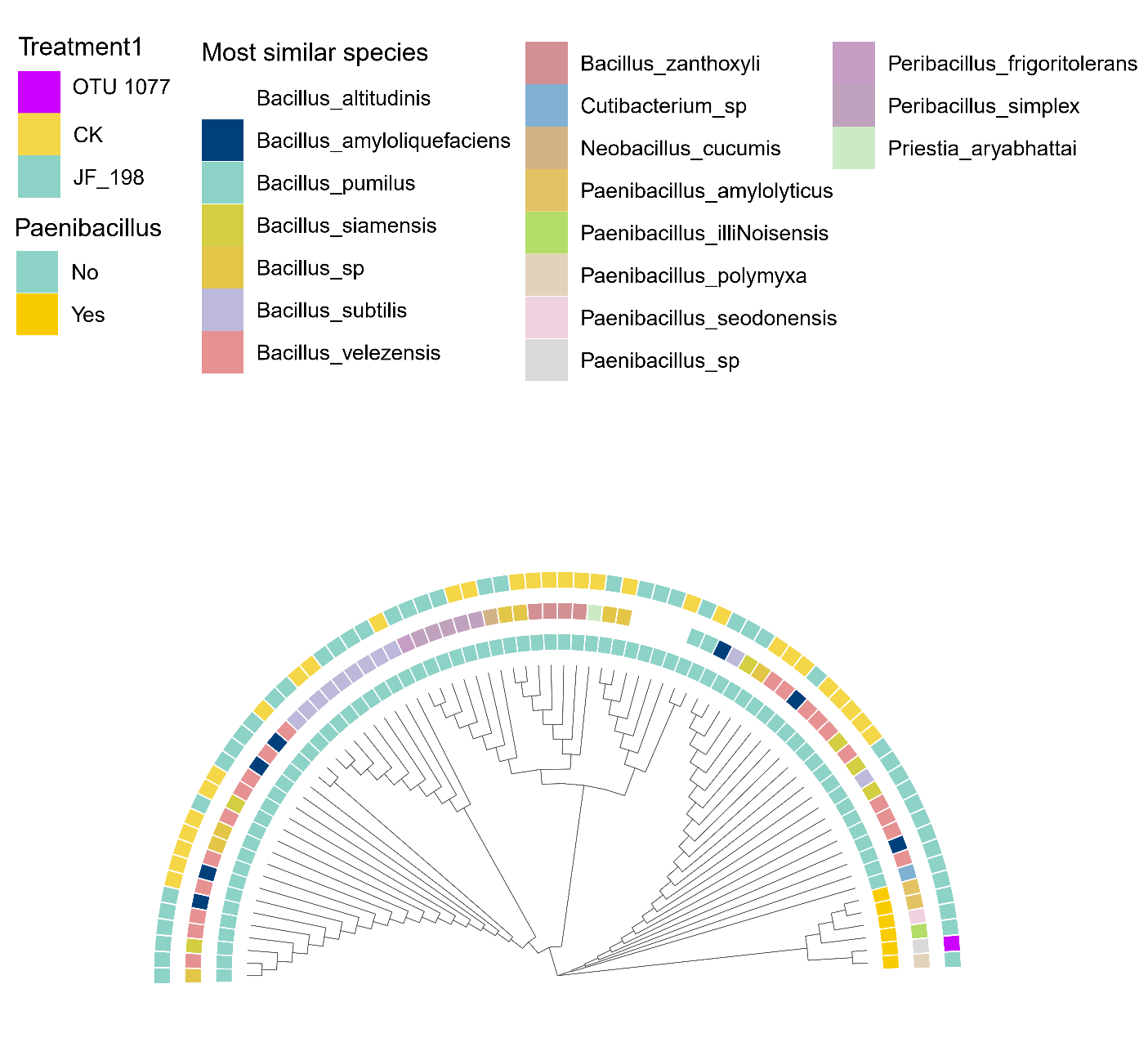

Figure S8 Cladogram illustrating the phylogenetic relationships among 76 rhizosphere soil Bacillales and OTU1077.
